# Recombinant antigen P29 of *Echinococcus granulosus* induces Th1, Tc1, and Th17 cell immune responses in sheep

**DOI:** 10.3389/fimmu.2023.1243204

**Published:** 2023-12-11

**Authors:** Jihui Yang, Yinqi Zhao, Yong Fu, Yongxue Lv, Yazhou Zhu, Mingxing Zhu, Jiaqing Zhao, Yana Wang, Changyou Wu, Wei Zhao

**Affiliations:** ^1^ Center of Scientific Technology of Ningxia Medical University, Yinchuan, China; ^2^ Ningxia Key Laboratory of Prevention and Treatment of Common Infectious Diseases of Ningxia Medical University, Yinchuan, China; ^3^ Qinghai Academy of Animal Sciences and Veterinary Medicine, Qinghai University, Xining, China; ^4^ School of Basic Medicine, Ningxia Medical University, Yinchuan, China; ^5^ Institute of Immunology, Zhongshan School of Medicine, Sun Yat-sen University, Guangzhou, China

**Keywords:** *Echinococcus granulosus*, recombinant antigen P29, Th1, Tc1, Th17, cellular immunity, sheep

## Abstract

*Echinococcosis* is a common human and animal parasitic disease that seriously endangers human health and animal husbandry. Although studies have been conducted on vaccines for *echinococcosis*, to date, there is no human vaccine available for use. One of the main reasons for this is the lack of in-depth research on basic immunization with vaccines. Our previous results confirmed that recombinant antigen P29 (rEg.P29) induced more than 90% immune protection in both mice and sheep, but data on its induction of sheep-associated cellular immune responses are lacking. In this study, we investigated the changes in CD4^+^ T cells, CD8^+^ T cells, and antigen-specific cytokines IFN-γ, IL-4, and IL-17A after rEg.P29 immunization using enzyme-linked immunospot assay (ELISPOT), enzyme-linked immunosorbent assay (ELISA), and flow cytometry to investigate the cellular immune response induced by rEg.P29 in sheep. It was found that rEg.P29 immunization did not affect the percentage of CD4^+^ and CD8^+^ T cells in peripheral blood mononuclear cells (PBMCs), and was able to stimulate the proliferation of CD4^+^ and CD8^+^ T cells after immunization *in vitro*. Importantly, the results of both ELISPOT and ELISA showed that rEg.P29 can induce the production of the specific cytokines IFN-γ and IL-17A, and flow cytometry verified that rEg.P29 can induce the expression of IFN-γ in CD4^+^ and CD8^+^ T cells and IL-17A in CD4^+^ T cells; however, no IL-4 expression was observed. These results indicate that rEg.P29 can induce Th1, Th17, and Tc1 cellular immune responses in sheep against *echinococcosis* infection, providing theoretical support for the translation of rEg.P29 vaccine applications.

## 1 Introduction

*Echinococcosis* is a common zoonotic parasitic disease, caused by the larvae of *Echinococcus* parasites in animals, including humans, which seriously endangers human health and has a substantial negative impact on the development of animal husbandry, contributing to huge expenses and economic losses in medicine and animal husbandry (1–4). Addressing *echinococcosis* requires two main components: prevention and clinical treatment (1). The clinical treatment phase is mainly for patients with encapsulated worm disease, and it includes both surgical and pharmacological forms of treatment. Patients are often in the middle and late stages of surgical treatment and have already suffered serious physical and psychological damage, which may also cause secondary infections and a high recurrence rate after surgery. Drug treatment has been effective, but there are problems such as the tendency to develop drug resistance and side effects, and encapsulated worm disease remains prevalent worldwide (5). Precisely because of the problems in clinical treatment, there is a great expectation for prevention to play a more effective role, therefore, vaccine research has received attention (6–8).

From traditional and genetic engineering vaccine development to nucleic acid, peptide, and multivalent vaccines, researchers have conducted substantial work in *echinococcosis* vaccine development and obtained better protective antigens such as Eg95 (9–12). The P29 protein, a 29 kDa antigen from *Echinococcus granulosus*, was first reported in 2000 by Gonza´lez et al (13). Our group conducted the first independent vaccine study of the P29 protein in China and successfully cloned and constructed the recombinant antigen P29 (rEg.P29). Immune protection of up to 96.6% was obtained in a mouse model of secondary infection (14). We also performed egg infection experiments in a sheep model, a suitable host for *E. granulosus*, and rEg.P29 was able to induce 94.8% immune protection (15). These findings demonstrate that the P29 protein is an excellent candidate molecule for *echinococcosis* vaccine. Although a series of studies have been conducted on *echinococcosis* vaccines, to date, no vaccine for human encysted worm disease has been officially used. The main reason for this is that there is insufficient research on its basic immunity. Moreover, numerous studies on the immunoprotective mechanisms of *echinococcosis* vaccines have used mice as animal models, while relatively few studies have been conducted using sheep as one of the most suitable disease hosts, which limits vaccine translation. Our previous study demonstrated that rEg.P29 induced high immune protection in sheep (15); however, little research on its mechanism of induced immune protection exists, especially research data on the induced cellular immune response in sheep.

In this study, based on our previous work, sheep were immunized with rEg.P29, and the rEg.P29-induced cellular immune response was studied using samples of peripheral blood and spleen and mesenteric lymph nodes. The aim of this study is to investigate the cellular immune response induced by rEg.P29 in sheep, to provide a theoretical basis for the development of vaccine applications, and to promote the use of the rEg.P29 vaccine in animals and humans.

## 2 Materials and methods

### 2.1 Preparation of rEg.P29

The recombinant plasmids containing P29 used in this study were stored in our laboratory. The expression and purification of rEg.P29 were performed according to our previously published method (16). Briefly, the strains were inoculated in LB liquid medium containing 0.1 mM isopropyl β-d-thiogalactoside (IPTG, Invitrogen, Waltham, USA) and incubated at 37°C for 10h. Then, rEg.P29 purification was performed according to the manufacturer’s instructions of the histidine-tagged protein purification kit (Merck, Kenilworth, USA). Endotoxin was removed from the antigen using an Endotoxin Removal Kit (Genscript, Nanjing, China). The purified rEg.P29 with endotoxin removed was identified by SDS-PAGE and the antigen concentration was determined using a BCA kit (KeyGenBiotech, Nanjing, China).

### 2.2 Animals and immunization

Thirty-six 4–6-month-old female Chinese Yan chi Tan sheep were randomly selected, and individual information is presented in **Supplementary Table 1**. Sheep were randomly divided into four groups of nine animals each, and each group was immunized according to three subcutaneous points (**Supplementary Figure 1**): the PBS control group was injected with 1 mL sterile PBS, the Quil A adjuvant control group was injected with 1 mg Quil A adjuvant solution, the rEg.P29 immunization group was injected with 50 μg rEg.P29, and the rEg.P29+Quil A immunization group was injected 50 μg rEg.P29 + 1 mg Quil A adjuvant. We conducted primary and booster immunizations at weeks 0 and 4, respectively.

### 2.3 Sample collection and cell culture

Peripheral blood was collected via the jugular vein at weeks 0, 4, 6, 8, 12 and 20, respectively. Sheep were euthanized at week 20 and spleens and mesenteric lymph nodes were collected for testing. Serum and peripheral blood mononuclear cells (PBMCs) were separated according to our previously published method (17). Briefly, anticoagulated peripheral blood was obtained by centrifugation at 1000 g for 10 min followed by aspiration of the upper layer. PBMCs were obtained by density gradient centrifugation, but without the buffy-coat stage. Whole blood was diluted 1:1 with PBS, mixed, and spread over an equal volume of lymphocyte isolate (Lymphoprep 1.087 g/L, TBD Science, Tianjin, China), centrifuged at 1130 g for 30 min at 22°C without brakes, and the white PBMCs layer was collected and washed twice. Sheep were euthanized by intravenous injection of 100 mg/kg potassium chloride under sedation with 20 mg/kg propofol intravenously. The spleen and mesenteric lymph nodes were harvested after euthanasia of the sheep, and the corresponding lymphocytes were isolated and obtained separately. The tissue was ground using a syringe piston and filtered through a 70-micron filter. The subsequent separation of lymphocytes was performed in the same way as for PBMCs. Cells were counted and then adjusted to 1×10^6^/mL using RPMI-1640 medium (GIBCO, Grand Island, USA) containing 10% fetal bovine serum (FBS), 2mM l-glutamine, 50 µM beta-mercaptoethanol 100 IU/mL penicillin, and 50 μg/mL streptomycin (Sigma-Aldrich, Saint Louis, USA) (18).

### 2.4 Flow cytometry analysis

To determine the cellular phenotypes of PBMCs, cells were directly stained with mouse anti-sheep CD4 (Clone: 44.38) and mouse anti-sheep CD8 (Clone: 38.65) antibodies (Bio-Rad AbD Serotec, Munich, USA), and incubated at 4°C for 30 min in the dark, followed by two washes with washing buffer. For intracellular cytokine assays, cells were incubated with or without rEg.P29 (10 μg/mL), and both had anti-CD28 (1 μg/mL) added at 37°C and 5% CO_2_ for 24 h. BFA (10 μg/mL) was added 6 h before the end of incubation. The cells were collected and washed twice with PBS containing 0.1% bovine serum albumin (BSA), blocked with 20% normal mouse serum, and stained with mouse anti-sheep CD4 (Clone: 44.38) and mouse anti-sheep CD8 (Clone: 38.65), and incubated at 4°C for 30 min in the dark, followed by two washes. Cells were fixed with 4% paraformaldehyde, washed twice, and permeabilized with PBS containing 0.1% BSA and 0.1% saponin overnight at 4°C. Cells were stained with mouse anti-bovine IFN-γ (Clone : CC302; Bio-Rad AbD Serotec, Munich, USA), mouse anti-bovine IL-4 (Clone : CC303; Bio-Rad AbD Serotec, Munich, USA), and mouse anti-human IL-17A(Clone : MT504; Mabtech, Nacka, Sweden) (three antibodies cross-reacted with sheep) at 4°C for 30 min in the dark. After cell washing, data were acquired using a BD FACSCelesta flow cytometer (Becton Dickinson, USA), and data analysis was performed using FlowJo software (Becton Dickinson, USA). Data were analyzed using fluorescence minus one (FMO) control for the gating strategy.

### 2.5 Enzyme-linked immunospot (ELISPOT)

Cells were adjusted to 1×10^6^/mL using RPMI-1640 medium. We used a bovine IFN-γ/IL-4/IL-17A ELISPOTPLUS kit (Mabtech, Nacka, Sweden) for detecting intracellular factor production according to the manufacturer’s instructions. ELISPOT plates were closed with RPMI-1640 medium containing 10% FBS, and 200 µL of cell suspension was added to each well with or without rEg.P29 (10 μg/mL) in the presence of anti-CD28 (1 μg/mL), then plates were incubated at 37°C with 5% CO_2_ for 24 h. After discarding the cell suspension, the plates were washed with PBS and the biotinylated detection antibody was added followed by Streptavidin-HRP. Finally, the plates were developed using TMB substrate and stopped using deionized water. The number of spots was read on an AID ELISPOT Reader Classic (Autoimmun Diagnostika Gmbh, Straßberg, Germany). Each group was set up in triplicate wells and the results are shown as their mean values.

### 2.6 Enzyme-linked immunosorbent assay (ELISA)

Cells were adjusted to 2×10^6^/mL using RPMI-1640 medium, and 200 µL of cell suspension was added to each well of a 96-well round bottom plate with or without rEg.P29 (10 μg/mL) in the presence of anti-CD28 (1 μg/mL) (19, 20). Plates were incubated at 37°C with 5% CO_2_ for 96 h, and the cytokine in the cell culture supernatant was quantified using the Bovine IFN-γ/IL-4 ELISA kit and Sheep IL-17A ELISA kit (Mabtech, Nacka, Sweden) according to the manufacturer’s instructions. The optical density of the plates was read at 450 nm using a Multiskan SkyHigh Microporous plate spectrophotometer (Thermo Fisher, Waltham, USA), and standard curves were generated to calculate cytokine concentrations.

### 2.7 Cell proliferation assay

The carboxyfluorescein diacetate succinimidyl ester (CFSE; Thermo Fisher, Waltham, USA) method was used to evaluate cell proliferation. CFSE was prepared using room temperature PBS to a concentration of 5μM, and PBMCs (1×10^7^/mL) were labeled with CFSE and incubated at 37°C with 5% CO_2_ for 15 min. The reaction was terminated by adding pre-cooled medium containing 10% FBS at 4°C for 5 min. Cells were washed with cold PBS and incubated with or without rEg.P29 (10 μg/mL) at 37°C with 5% CO_2_ for 96 h. Cells were collected and labeled with antibodies, and data were collected using a BD FACSCelesta flow cytometer and analyzed using FlowJo software.

### 2.8 Statistical analysis

All results were analyzed using SPSS 22.0 (GraphPad Software Inc., San Diego, USA) and GraphPad Prism 8.0 (IBM Corp., Armonk, NY, USA). For comparing two groups, Mann-Whitney test was used, and one-way ANOVA was used for comparing three or more groups. Data are shown as mean or mean ± standard deviation (SD). Differences were considered statistically significant when *P* < 0.05.

## 3 Results

### 3.1 rEg.P29 does not affect CD4^+^ and CD8^+^ T cells in PBMCs by flow cytometry

**Figure 1:**
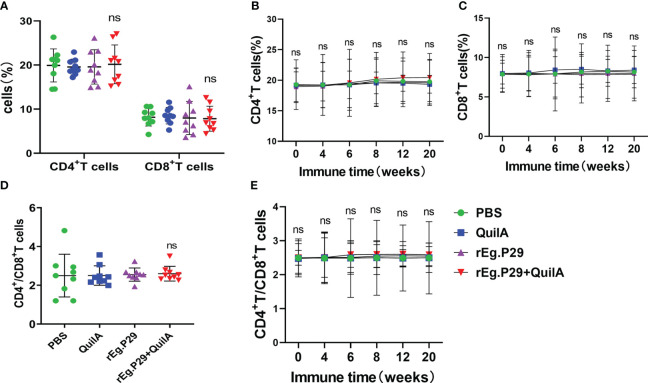
CD4^+^ and CD8^+^ T cells in PBMCs. Peripheral blood samples were collected at different immunization times, and CD4^+^ and CD8^+^ T cells in PBMCs were analyzed using flow cytometry. **(A)** Proportion of CD4^+^ and CD8^+^ T cells in PBMCs of each group at week 8. **(B, C)** show the trend of the proportion of CD4^+^ and CD8^+^ T cells at different times, respectively. **(D)** The ratio of CD4^+^ to CD8^+^ T cells in each group of PBMCs at week 8. **(E)** The trend of the ratio of CD4^+^ to CD8^+^ T cells at different times. Data were obtained from 9 sheep, and results are presented as mean ± SD (*ns*, *P* > 0.05).

Peripheral blood samples were collected from each immunization group at different immunization times, and CD4^+^ and CD8^+^ T cells in PBMCs were analyzed using flow cytometry. Consistent with other animals, CD4^+^ and CD8^+^ T cells were relatively stable, and the percentage of CD4^+^ T cells was significantly higher than that of CD8^+^ T cells (**Figure 1A**). After immunization with rEg.P29 supplemented with adjuvant, there was a tendency for CD4^+^ T cells and CD4^+^/CD8^+^ T cells to increase compared with other immunization groups, especially CD4^+^ T cells, but none of these results were statistically significant (**Figures 1B, D, E**). rEg.P29 had no effect on CD4^+^ and CD8^+^ T cells in PBMCs, but the trend indicates that it has the potential to enhance immunity.

### 3.2 rEg.P29 stimulates the production of antigen-specific cytokines IFN-γ and IL-17A by ELISPOT and ELISA

**Figure 2:**
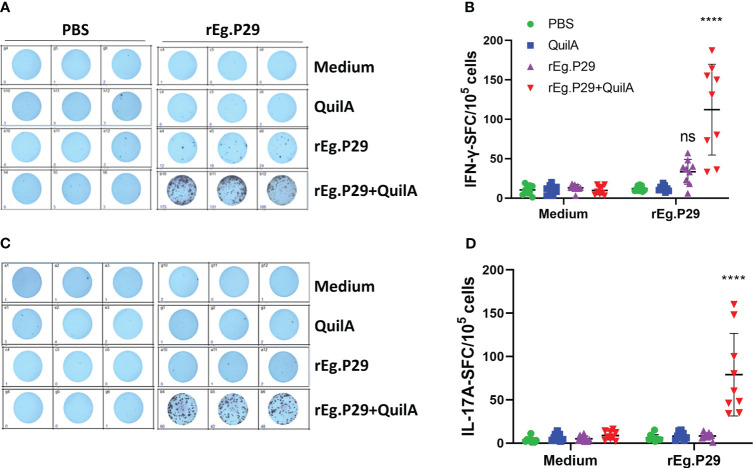
Cytokines produced in PBMCs by ELISPOT. After stimulation of PBMCs by rEg.P29 *in vitro*, cells producing the specific cytokines IFN-γ and IL-17A were analyzed using ELISPOT, and the results are shown as spot-forming cells (SFC) (samples at week 8). **(A, B)** show antigen-specific IFN-γ SFC. **(C, D)** show antigen-specific IL-17A SFC. Representative images of each cytokine spot-forming cell are shown in the figure. Data were obtained from 9 sheep, and results are presented as mean ± SD (****P < 0.0001; *ns*, *P* > 0.05).

ELISPOT assays can sensitively detect cells expressing specific cytokines, and to observe vaccine-induced specific cytokines, we first measured cells secreting specific cytokines IFN-γ, IL-4, and IL-17A under stimulation with rEg.P29, and the results were shown as spot-forming cells (SFC). The number of cells secreting IFN-γ was significantly higher in the rEg.P29+Quil A group compared to the other immunization groups, and we found that the rEg.P29 immunization group also produced a number of IFN-γ-secreting cells, but without statistical differences (**Figures 2A, B**). In the IL-17A ELISPOT assay, the number of IL-17A-secreting cells in the rEg.P29+Quil A group was also significantly higher than in the other groups (**Figures 2C, D**). The IL-4 ELISPOT assay showed that IL-4-secreting cells were not found in any group (Data not presented).

**Figure 3:**
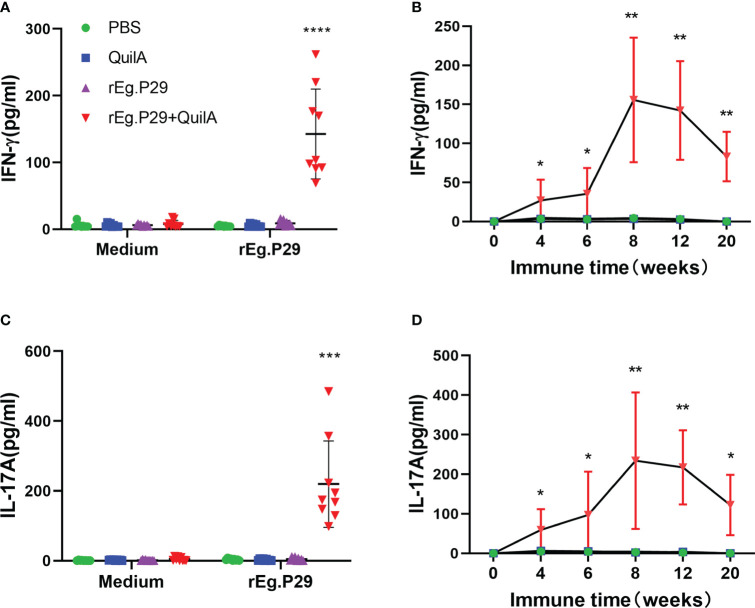
Cytokines produced in PBMCs by ELISA. After rEg.P29 stimulation of PBMCs, the level of three antigen-specific cytokines in the culture supernatant was quantified using ELISA. **(A)** Antigen-specific IFN-γ levels in the culture supernatant of PBMCs samples at week 8. **(B)** Trends of antigen-specific IFN-γ levels in PBMCs culture supernatants of samples at different times. **(C)** Antigen-specific IL-17A levels in the culture supernatant of PBMCs samples at week 8. **(D)** Trends of antigen-specific IL-17A levels in PBMCs culture supernatants of samples at different times. Data were obtained from 9 sheep, and results are presented as mean ± SD (*P < 0.05; **P < 0.01; ***P < 0.001; ****P < 0.0001).

We also measured the levels of these three antigen-specific cytokines in the cell culture supernatants of each group at different immunization times using an ELISA. No IFN-γ was produced in the PBS, Quil A, or rEg.P29 groups, while a high level of IFN-γ was produced in the cell supernatant of the rEg.P29+Quil A group, which was significantly higher than the other three immunization groups (**Figure 3A**). The time-varying results of specific IFN-γ showed that IFN-γ gradually increased in the rEg.P29+QuilA group, peaking at week 8, and then gradually decreased, but remained significantly higher than other groups, where specific IFN-γ production was consistently not detected (**Figure 3B**). The ELISA results and temporal patterns of the specific cytokine IL-17A in culture supernatants were similar to those of IFN-γ. Relatively high levels of IL-17A were produced only in the rEg.P29+QuilA group, but not in the other groups (**Figures 3C, D**). The specific cytokine IL-4 was consistently not detected in the cell culture supernatant of all groups (Data not presented).

**Figure 4:**
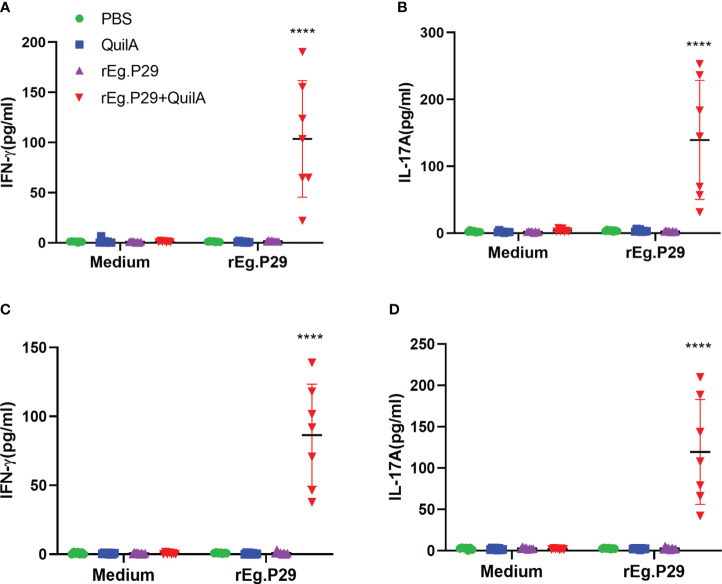
Cytokines produced in lymphocytes of spleen and mesenteric lymph nodes by ELISA analysis. Sheep were euthanized and spleen lymphocytes were obtained. The cells were stimulated with rEg.P29 and the levels of three antigen-specific cytokines in the culture supernatant were quantified using ELISA. **(A, B)** show the antigen-specific IFN-γ and IL-17A levels in the supernatants of spleen, respectively. **(C, D)** show the antigen-specific IFN-γ and IL-17A levels in the supernatants of mesenteric lymph nodes, respectively. Data were obtained from 7 sheep, and results are presented as mean ± SD (****P < 0.0001).

Sheep were euthanized and the results of the three cytokine levels in the culture supernatants of lymphocytes isolated from the spleen and mesenteric lymph nodes were consistent with the ELISA results of PBMCs, with both indicating that rEg.P29 induced the production of IFN-γ and IL-17A (**Figure 4**), but not IL-4 (Data not presented). The results of ELISPOT and ELISA showed that rEg.P29 induced the production of the antigen-specific cytokines IFN-γ and IL-17A in sheep to protect against *E.granulosus* infection.

### 3.3 rEg.P29 induces Th1, Tc1, and Th17 cellular immune responses by Flow cytometry

**Figure 5:**
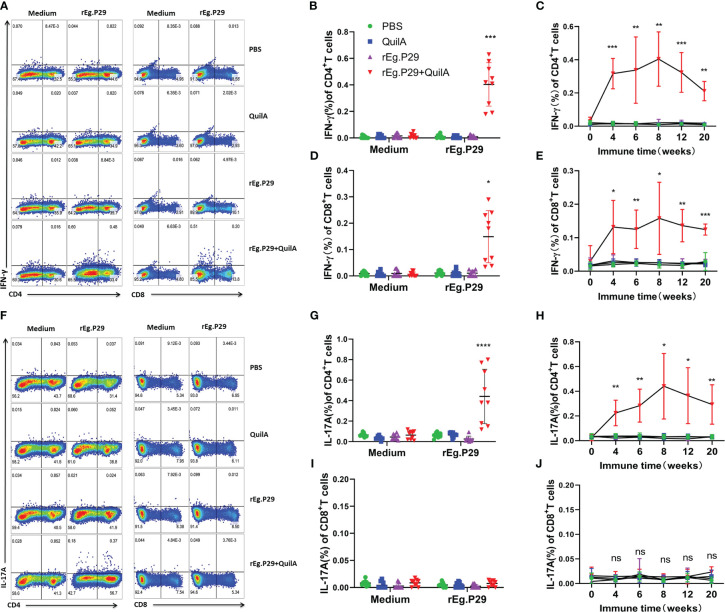
Cytokines produced in PBMCs by flow cytometry. PBMCs were stimulated with rEg.P29, and the cells were collected and labeled with antibodies to detect IFN-γ and IL-17A production by CD4^+^ and CD8^+^ T cells using flow cytometry. **(A)** Representative flow scatter plots for detecting IFN-γ production by CD4^+^ and CD8^+^ T cells. **(B, D)** show the IFN-γ production by CD4^+^ and CD8^+^ T cells in each group at week 8, respectively. **(C, E)** show the tendency of IFN-γ production by CD4^+^ and CD8^+^ T cells at different times, respectively. **(F)** Representative flow scatter plots for detecting IL-17A production by CD4^+^ and CD8^+^ T cells. **(G, I)** show the IL-17A production by CD4^+^ and CD8^+^ T cells in each group of samples at week 8, respectively. **(H, J)** show the tendency of IL-17A production by CD4^+^ and CD8^+^ T cells at different times, respectively. Data were obtained from 9 sheep, and results are presented as mean ± SD (*P < 0.05; **P < 0.01; ***P < 0.001; ****P < 0.0001).

ELISPOTs and ELISAs are capable of highly sensitive, quantitative detection of cytokines; however, they cannot determine which population of cells produced the cytokines. To further identify which group of cells produced IFN-γ and IL-17A, cells were stimulated *in vitro* by antigen, and the cytokine expression in CD4^+^ and CD8^+^ T cells was detected using flow cytometry. The expression of IFN-γ in both CD4^+^ and CD8^+^ T cells was significantly higher in PBMCs of the rEg.P29+QuilA group after antigen stimulation than in the other groups, and the level of IFN-γ in CD4^+^ T cells was higher than that in CD8^+^ T cells (**Figures 5A, B, D**). Moreover, the cytokine IFN-γ in CD4^+^ and CD8^+^ T cells gradually increased over time and peaked at week 8, after which it gradually decreased but remained at a high level, always significantly higher than the other groups (**Figures 5C, E**). High levels of antigen-specific IL-17A expression were also detected in CD4^+^ T cells, but IL-17A was not produced in CD8^+^ T cells (**Figures 5F, G, I, J**). In addition, the level of IL-17A in CD4^+^ T cells changed over time in a manner consistent with that of IFN-γ in CD4^+^ T cells (**Figure 5H**).

**Figure 6:**
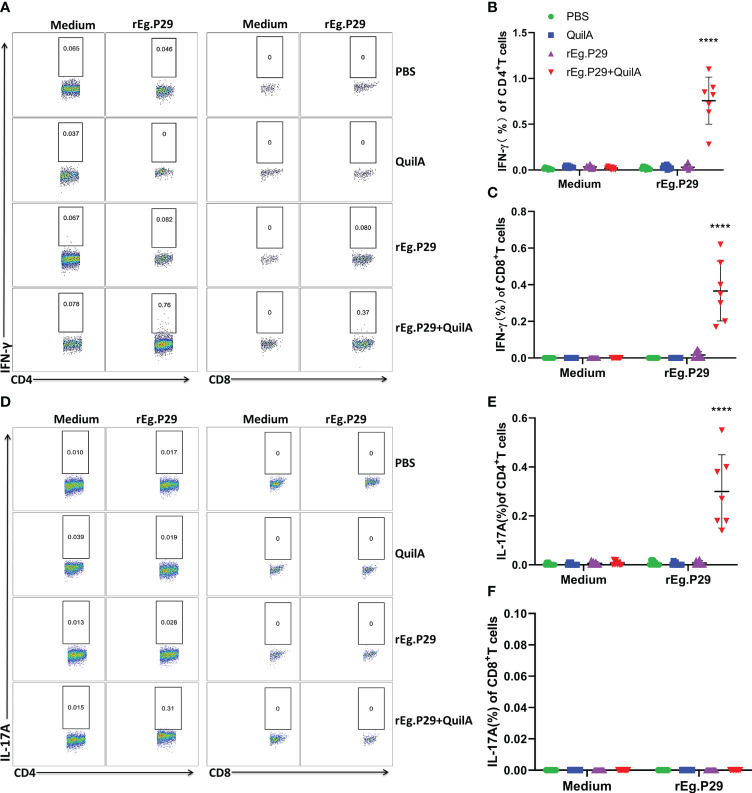
Cytokines produced in spleen lymphocytes by flow cytometry. Spleen lymphocytes were obtained after euthanasia of sheep, cells were labeled with antibodies after rEg.P29 stimulation, and IFN-γ and IL-17A production by CD4^+^ and CD8^+^ T cells was detected using flow cytometry. **(A)** Representative flow scatter plots for detecting IFN-γ production by CD4^+^ and CD8^+^ T cells. **(B, C)** show the IFN-γ production by CD4^+^ and CD8^+^ T cells in each group, respectively. **(D)** Representative flow scatter plots for detecting IL-17A production by CD4^+^ and CD8^+^ T cells. **(E, F)** show the IL-17A production by CD4^+^ and CD8^+^ T cells in each group, respectively. Data were obtained from 7 sheep, and results are presented as mean ± SD (****P < 0.0001).

**Figure 7:**
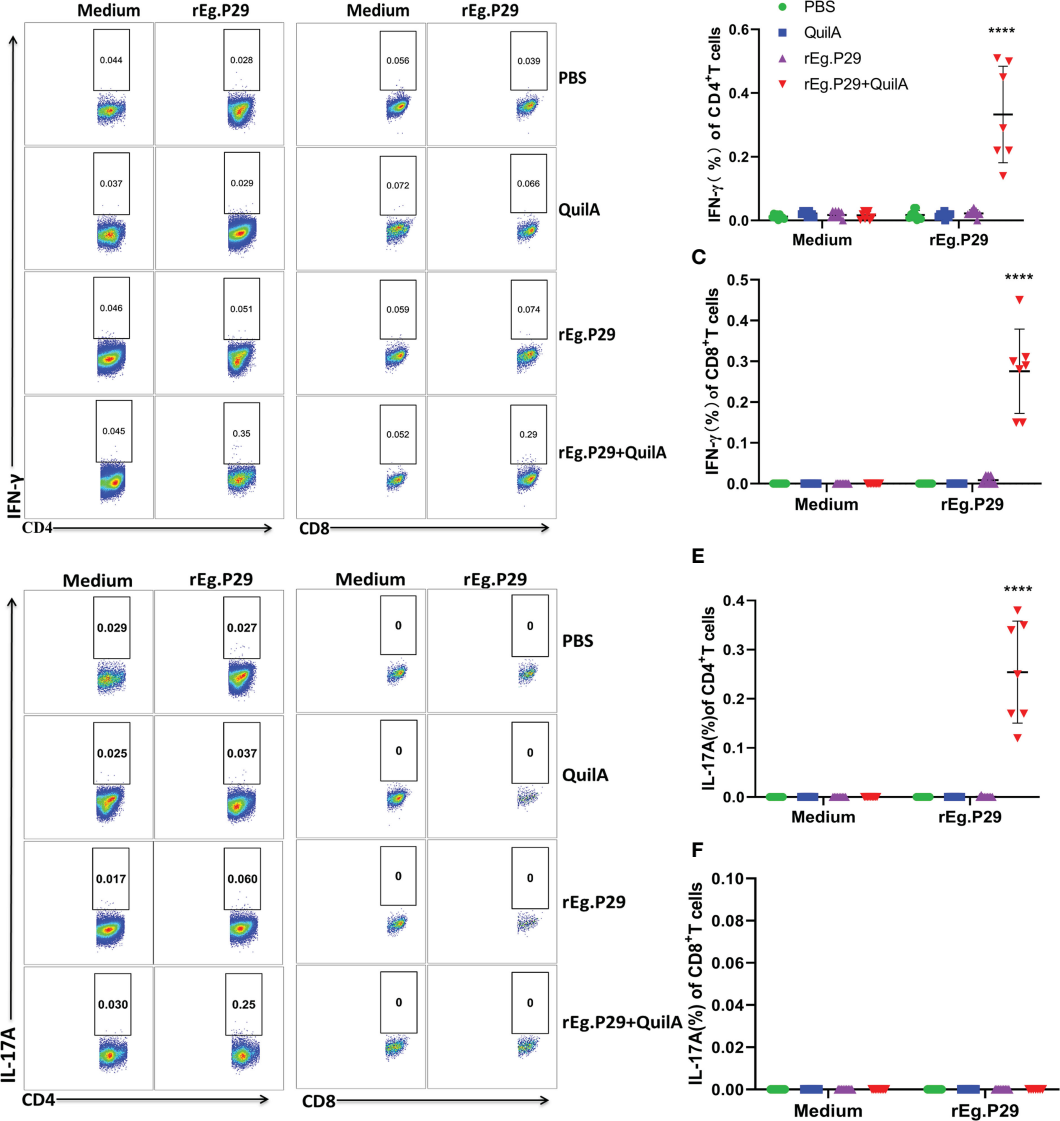
Cytokines produced in lymphocytes of mesenteric lymph nodes by flow cytometry. Lymphocytes of mesenteric lymph nodes were obtained after euthanasia of sheep, cells were labeled with antibodies after rEg.P29 stimulation, and IFN-γ, IL-4, and IL-17A production by CD4^+^ and CD8^+^ T cells was detected using flow cytometry. **(A)** Representative flow scatter plots for detecting IFN-γ production by CD4^+^ and CD8^+^ T cells. **(B, C)** show the IFN-γ production by CD4^+^ and CD8^+^ T cells in each group, respectively. **(D)** Representative flow scatter plots for detecting IL-17A production by CD4^+^ and CD8^+^ T cells. **(E, F)** show the IL-17A production by CD4^+^ and CD8^+^ T cells in each group, respectively. Data were obtained from 7 sheep, and results are presented as mean ± SD (****P < 0.0001).

We also examined cytokine expression in CD4^+^ and CD8^+^ T cells after *in vitro* culturing of lymphocytes from the spleen and mesenteric lymph nodes stimulated with rEg.P29. The findings were consistent with those of PBMCs which expressed the specific cytokines IFN-γ and IL-17A in CD4^+^T cells, and IFN-γ in CD8^+^T cells (**Figures 6A–C**, **7A–C**, **6D–F**, **7D–F**). For the IL-4-expressing cell population, none were detected in lymphocytes from PBMCs, the spleen, or the mesenteric lymph nodes, which is consistent with the ELISPOT and ELISA results (Data not presented). These results strongly suggest that rEg.P29 supplemented with adjuvant-induced sustained Th1, Tc1, and Th17 cellular immune responses in sheep.

### 3.4 rEg.P29 stimulates proliferation of CD4^+^ and CD8^+^ T cells by flow cytometry

**Figure 8:**
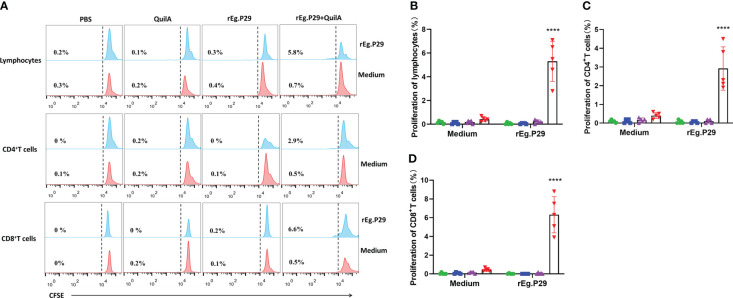
Proliferation of CD4^+^ and CD8^+^ T cells. PBMCs were labeled with CFSE, stimulated *in vitro* with rEg.P29, and the decrease in CFSE fluorescence intensity of labeled cells was detected using flow cytometry to assess the proliferation of CD4^+^ and CD8^+^ T cells. **(A)** Histogram plots of CFSE fluorescence of lymphocytes, CD4^+^, and CD8^+^ T cells. **(B–D)** represent the proliferation frequencies of lymphocytes, CD4^+^, and CD8^+^ T cells, respectively. Data were obtained from 5 sheep, and results are presented as mean ± SD (****P < 0.0001).

PBMCs from each group were labeled with CFSE, stimulated *in vitro* with or without rEg.P29, and CD4^+^ and CD8^+^ T cell proliferation was assessed by detecting the decrease in CFSE fluorescence intensity of labeled cells using flow cytometry. The CFSE intensity of lymphocytes, CD4^+^ and CD8^+^ T cells in the rEg.P29+QuilA group decreased in the presence of rEg.P29, indicating that significant cell proliferation occurred; however, no cell proliferation occurred in the absence of rEg.P29 (**Figure 8**). Moreover, the proliferation of CD8^+^ T cells stimulated by rEg.P29 was higher than that of CD4^+^ T cells (**Figures 8C, D**).

## 4 Discussion

rEg.P29 is a vaccine antigen with good potential for protection against *echinococcosis*. Our previous results demonstrated that rEg.P29 induced strong and sustained humoral and cellular immune responses in mice, and obtained up to 96.6% immune protection in a mouse model of secondary infection with *E. prowazekii* (14, 21, 22). Moreover, rEg.P29 also induced a sustained humoral immune response in sheep, inducing 94.8% immune protection (15). However, the lack of research data on the induction of cellular immunity by rEg.P29 has somewhat limited the promotion of its application. In the present study, we selected 36 female Chinese Yan chi Tan sheep of 4-6 months of age, similar body weight, and negative for antibodies against *echinococcosis*, to study the cellular immune response induced by rEg.P29.

T cells play a vital role in controlling pathogenic infections and clearing pathogens, with CD4^+^ T cells (Th cells) and CD8^+^ T cells (Tc cells) playing important roles (23, 24). CD4^+^ T cells recognize MHC-II antigen molecules, mainly by promoting antigen presentation and upregulating co-stimulatory molecules on dendritic cells to induce a strong CD8^+^ T cell response (25, 26). CD8^+^ T cells recognize MHC-I molecules and can differentiate into cytotoxic cells capable of resisting pathogenic invasion (27, 28). Treatment of chronic Toxoplasma gondii infection with antigen-specific undepleted CD4^+^ T cells restores CD8^+^ T cell function in the host and prevents potential reinfection, suggesting that CD8^+^ T cells require the help of CD4^+^ T cells (29). The level of immunity or immune status of the body can be reflected to some extent by the ratio of CD4^+^ to CD8^+^ T cells (30). The skin and local lymph of sheep and goats show strong immune and inflammatory responses to pustular dermatitis virus infection, exhibiting cellular immune responses including CD4^+^ and CD8^+^ T cell changes (31). Increased CD4^+^ and CD8^+^ T cells in the primary immune response to bluetongue virus infection in sheep trigger a T-cell response to resist viral infection (32–34). Baron et al. showed that specific CD4^+^ T cells are the main mediators protecting the host against viral infection in a study on peste des petits ruminant’s virus infection in goats (35). Our examination of the ratios of CD4^+^ and CD8^+^ T cells in PBMCs from samples at different immunization times showed that the ratio of CD4^+^ T cells was significantly higher than that of CD8^+^ T cells in all groups. After immunization with rEg.P29 co-adjuvant, the CD4^+^ T cell ratio and CD4^+^/CD8^+^ T cells tended to increase, gradually stabilizing with the extension of immunization time; however, they did not differ, which may be related to the limited number of samples and the large individual differences between sheep. This also reflects the possibility that rEg.P29 immunization can enhance the sheep’s immunity level and facilitate protection against pathogenic infections.

Upon encountering specific antigens on antigen-presenting cells, CD4^+^ T cells clonally expand and differentiate into different cell subpopulations with different functions. These cell subsets include T helper 1 (Th1), Th2, Th17, and T follicular helper (Tfh) cells that coordinate immune responses to different microbial pathogens (36, 37). This study focused on the *in vitro* stimulation of PBMCs, splenic lymphocytes, and mesenteric lymph node lymphocytes from post-P29-immunization samples and detected antigen-specific IFN-γ, IL-4, and IL-17A production using ELISPOT, ELISA, and flow cytometry immunological methods. We observed the dynamic changes of antigen-specific cytokines to evaluate the induced Th1, Th2, and Th17 cellular immune responses. The proliferation of each lymphocyte population after antigen stimulation was examined using flow cytometry, and the results showed that rEg.P29 could stimulate the proliferation of antigen-specific lymphocytes. rEg.P29 co-adjuvant immunization was effective in inducing sustained Th1, Th17, and Tc1 cellular immune responses in sheep, and no Th2 response was detected. Clinical studies have shown that effective anti-*echinococcal* tapeworm infection treatment is associated with a Th1 response, while ineffective anti-infection treatment is strongly associated with elevated Th2-type cytokine expression (38). In the chronic phase of *E. granulosus* infection, Th1 and Th2 immune responses coexist and their balance plays a key role in parasite immune tolerance and evasion. Mouse models of cystic *echinococcosis* infection showed that the early stage of infection was mainly characterized by increased expression levels of Th1 cytokines, including IFN-γ and TNF-α, which could inhibit or kill the parasite *in vivo*. In the late stage of infection, the Th1-type response gradually shifts to a Th2-type response and contributes to the expression of anti-inflammatory cytokines, which facilitates the survival of the parasite (39, 40). Ma et al. found that rBCG-EgG1Y162 protected mice from infection with *E. granulosus*, with an important role played by the elevated IFN-γ and IL-2 cellular immune response (41). Recombination of *E. granulosus* Eg95 with recombinant rabies virus LBNSE produced an Eg95-specific IFN-γ Th1 cell response and induced more than 90% protection in mice after immunization (42). The Th1-type cellular immune response induced by rEg.P29 in sheep in this study is consistent with the results of the above-mentioned related studies.

Th17 cells, a newly discovered cell type in recent years, play an immunoprotective role against *E. granulosus* infection by producing inflammatory cytokines (43–45). IL-17A belongs to one of the most important members of the IL-17 family and is mainly expressed in Th17 cells. Th17 cells that secrete IL-17A are closely associated with, among others, parasitic infections, and cancer treatment (46, 47). During helminth infection, the Th17 immune response regulates the host immune system and influences the severity of disease (48), with elevated Th17 expression observed in the early stages of *E. granulosus* infection (49, 50). As protective immunity against helminth infection, Th17 cells also play a complementary role to that of Th1 cells (51). Labsi et al. found that intravenous injection of recombinant IL-17A antibody was able to reduce the growth of protozoa by more than 90% and reduce the infection rate by two-thirds in mice (49). It has been shown that Th1 and Th17 type cytokines predominate in patients with hepatic cystic *echinococcosis* with inactive cysts, while Th2 type cytokines are more pronounced in patients with hepatic cystic *echinococcosis* with active cysts (52). Our study demonstrates that rEg.P29 can consistently induce a Th17-type cellular immune response in sheep, providing evidence for the immunoprotective effect of P29 against *E. granulosus*. Sad et al. define CD8^+^ T cells secreting the Th1-type cytokine IFN-γ as Tc1 cells, which are killer T cells with the ability to kill cells and play an important role not only in certain inflammatory responses, but also in autoimmune diseases (53–55). In this study, rEg.P29 also induced an immune effect in Tc1 cells in sheep, which was not found in our previous experimental studies in mice. This certainly adds to the evidence for rEg.P29-induced immune effects.

Sheep are one of the most suitable intermediate hosts for *E. granulosus* (4), and the study of the immune protection mechanism using sheep has important practical significance for the development and application of *E. granulosus* vaccines (56). In this study, we confirmed that rEg.P29 can effectively induce persistent and strong Th1, Th17, and Tc1 cellular immune responses in sheep. These findings provide theoretical support to promote the application of the rEg.P29 vaccine, accelerate its use in livestock producers, and may contribute to the development of human vaccines. There are some potential limitations of this study (small sample size, variability in individual sheep, etc.), which suggest that we should consider these factors in advance when using sheep as experimental animals.

## 5 Conclusion

rEg.P29 can induce Th1, Th17, and Tc1 cellular immune responses in sheep against *echinococcosis* infection and has good vaccine potential, providing theoretical support for the vaccine applications.

## Data availability statement

The original contributions presented in the study are included in the article/**Supplementary Material**. Further inquiries can be directed to the corresponding author.

## Ethics statement

The animal study was approved by Ethics Committee of Ningxia Medical University. The study was conducted in accordance with the local legislation and institutional requirements.

## Author contributions

JY and YiZ contributed equally to this study. CW and WZ designed the experiment. YL, YiZ, MZ, YF, and JZ collected animal samples. YL performed ELISPOT and ELISA assays. JY and YaZ performed flow cytometry. JY wrote and revised the manuscript. All authors contributed to the article and approved the submitted version.
