# Supplementary figures and images for: Recombinant antigen P29 of Echinococcus granulosus induces Th1, Tc1, and Th17 cell immune responses in sheep
Source: Front Immunol. 2023 Dec 11;14:1243204. doi: 10.3389/fimmu.2023.1243204 (PMC10768560; doi:10.3389/fimmu.2023.1243204)

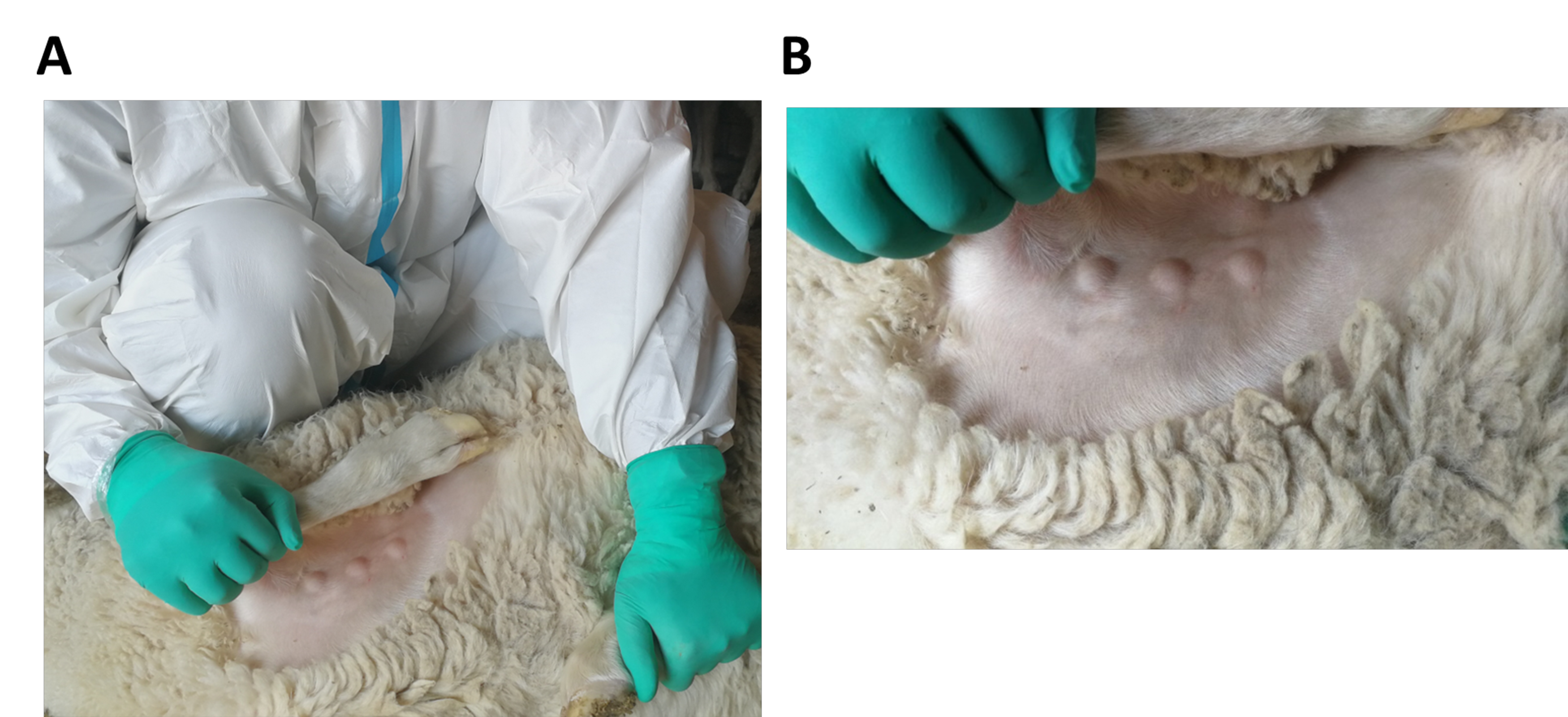

Supplement: Supplementary file 1 [file Image_1.tif]
