# Supplementary material for: Recombinant antigen P29 of Echinococcus granulosus induces Th1, Tc1, and Th17 cell immune responses in sheep
Source: Front Immunol. 2023 Dec 11;14:1243204. doi: 10.3389/fimmu.2023.1243204 (PMC10768560; doi:10.3389/fimmu.2023.1243204)
Supplement: Supplementary file 2 [file Table_1.docx]

**Table 1 Individual sheep information in this study**

| **No.** | **Species** | **Gender** | **Age** | **Weight(Kg)** | ***Echinococcosis*** |
| --- | --- | --- | --- | --- | --- |
| 1 | Chinese Yan chi Tan sheep  (the same below) | Female  (the same below) | 4-6 months  (the same below) | 25.2 | - |
| 2 |  |  |  | 22.4 | - |
| 3 |  |  |  | 20.4 | - |
| 4 |  |  |  | 27.5 | - |
| 5 |  |  |  | 22.7 | - |
| 6 |  |  |  | 22.8 | - |
| 7 |  |  |  | 23.8 | - |
| 8 |  |  |  | 20.4 | - |
| 9 |  |  |  | 23.0 | - |
| 10 |  |  |  | 21.1 | - |
| 11 |  |  |  | 21.0 | - |
| 12 |  |  |  | 23.8 | - |
| 13 |  |  |  | 21.9 | - |
| 14 |  |  |  | 25.7 | - |
| 15 |  |  |  | 26.0 | - |
| 16 |  |  |  | 22.4 | - |
| 17 |  |  |  | 25.7 | - |
| 18 |  |  |  | 25.8 | - |
| 19 |  |  |  | 23.8 | - |
| 20 |  |  |  | 18.2 | - |
| 21 |  |  |  | 23.2 | - |
| 22 |  |  |  | 19.8 | - |
| 23 |  |  |  | 19.8 | - |
| 24 |  |  |  | 18.3 | - |
| 25 |  |  |  | 30.5 | - |
| 26 |  |  |  | 22.7 | - |
| 27 |  |  |  | 24.3 | - |
| 28 |  |  |  | 24.0 | - |
| 29 |  |  |  | 23.5 | - |
| 30 |  |  |  | 30.5 | - |
| 31 |  |  |  | 20.9 | - |
| 32 |  |  |  | 19.0 | - |
| 33 |  |  |  | 24.0 | - |
| 34 |  |  |  | 24.7 | - |
| 35 |  |  |  | 21.2 | - |
| 36 |  |  |  | 18.0 | - |
